# ZnO Nanowire‐Based Early Detection of SARS‐CoV‐2 Antibody Responses in Asymptomatic Patients with COVID‐19

**DOI:** 10.1002/admi.202102046

**Published:** 2022-02-05

**Authors:** Jung Kim, Sung Kyun Lee, Jong‐Hwan Lee, Hye‐Yeon Kim, Nam Hoon Kim, Chang Hoon Lee, Chang‐Seop Lee, Hong Gi Kim

**Affiliations:** ^1^ Center for Convergent Research of Emerging Virus Infection Korea Research Institute of Chemical Technology Daejeon 34114 Republic of Korea; ^2^ Research Center for Bioconvergence Analysis Korea Basic Science Institute Cheonju 28119 Republic of Korea; ^3^ Drug Discovery Platform Research Center Therapeutic & Biotechnology Division Korea Research Institute of Chemical Technology Daejeon 34114 Republic of Korea; ^4^ Department of Internal Medicine Jeonbuk National University Medical School Jeonju Jeollabuk‐do 54986 Republic of Korea; ^5^ Biomedical Research Institute of Jeonbuk National University Hospital Jeonju Jeollabuk‐do 54907 Republic of Korea

**Keywords:** antibody response, asymptomatic, COVID‐19, early detection, ZnO nanowire

## Abstract

A serological immunoassay based on enzyme‐linked immunosorbent assay (ELISA) is a crucial tool for screening and identification of human SARS‐CoV‐2 seroconversion. Various immunoassays are developed to detect the spike 1 (S1) and nucleocapsid (NP) proteins of SARS‐CoV‐2; however, these serological tests have low sensitivity. Here, a novel microplate (MP) is developed on which a ZnO nanowire (NW) is fabricated by a modified hydrothermal synthesis method. This plate is coated with SARS‐CoV‐2 NP and used as a fluorescent immunoassay (FIA) to detect antibodies specific for SARS‐CoV‐2 NP. Compared with the bare MP, the ZnO‐NW MP binds high levels (up to 5 µg mL^−1^) of SARS‐CoV‐2 NP tagged to histidine without any surface treatment. A novel serological assay based on the ZnO‐NW MP is more sensitive than a commercial immunoassay, enabling early detection (within <5 days of a reverse transcription polymerase chain reaction‐confirmed COVID‐19 infection) of anti‐SARS‐CoV‐2 NP IgG antibodies in asymptomatic patients with COVID‐19. This is the first assay to detect early antibody responses to SARS‐CoV‐2 in asymptomatic patients. Therefore, this serological assay will facilitate accurate diagnosis of COVID‐19, as well as estimation of COVID‐19 prevalence and incidence.

## Graphical abstract

A novel microplate (MP) on which a ZnO nanowire (NW) is fabricated by a modified hydrothermal synthesis method to detect antibodies specific for SARS‐CoV‐2 NP antigen is presented. A novel serological assay based on the ZnO‐NW MP is more sensitive than a commercial immunoassay, enabling early detection of anti‐SARS‐CoV‐2 NP IgG antibodies in asymptomatic patients with COVID‐19.

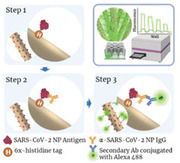

## 1 Introduction

Coronavirus disease 2019 (COVID‐19), caused by severe acute respiratory syndrome coronavirus 2 (SARS‐CoV‐2), first appeared in Wuhan city, Hubei province, China, in December 2019, and the pandemic is ongoing.^[^ ^1^ ^]^ Infection by SARS‐CoV‐2 causes pneumonia, with symptoms such as fever, fatigue, and cough.^[^ ^2^ ^]^ Despite development of new vaccines and identification of antiviral drugs, transmission of SARS‐CoV‐2 remains high.^[^ ^3^ ^]^ Molecular diagnosis using reverse transcription‐quantitative polymerase chain reaction (RT‐qPCR) is the gold standard for accurate detection of the virus in symptomatic and asymptomatic patients; however, the RT‐PCR test is labor‐intensive and expensive.^[^ ^4^ ^]^ Undocumented cases and asymptomatic infections are associated with continued high rates of transmission.^[^ ^5^ ^]^ Asymptomatic patients with COVID‐19 showed significantly lower level of the virus‐specific IgG levels compared to the symptomatic group.^[^ ^6^ ^]^ For that reason, immunological testing to detect antibodies specific for viral antigens is important to get a more accurate estimate of the prevalence and incidence of SARS‐CoV‐2.

Serology tests based on enzyme‐linked immunosorbent assays (ELISA) and immunochromatographic lateral flow assays (LFA) can detect antibodies specific for the spike (S) and nucleocapsid (NP) proteins of SARS‐CoV‐2.^[^ ^7^, ^8^ ^]^ The LFA test is quick, easy, and cost‐effective, but has a relatively low sensitivity. The ELISA test is the gold standard, high throughput immunological method of detecting antibodies.^[^ ^9^ ^]^ Various ELISA tests have been used for detection of anit‐COVID‐19 antibodies.^[^ ^10^ ^]^ Individuals who have recovered from SARS‐CoV‐2 infection maintain antibodies for some time.^[^ ^11^, ^12^ ^]^ However, a recent study reported that immunochromatographic tests have low sensitivity (3.7%) for immunoglobin G (IgG) produced at the early stage (<7 days) of COVID‐19 infection (*n* = 27).^[^ ^13^ ^]^ Another study reported development of a sensitive assay based on fluorescence‐mediated detection of seroconversion in COVID‐19 patients after symptom onset.^[^ ^14^ ^]^ However, there are no reports of serological tests that can detect immunoglobulins at an early stage in asymptomatic patients infected with SARS‐CoV‐2.

There have been many attempts to increase the reaction area of nanostructures by changing the surface morphology.^[^ ^15^, ^16^, ^17^, ^18^, ^19^, ^20^, ^21^, ^22^, ^23^, ^24^, ^25^, ^26^ ^]^ Application of nanostructures to biological reactions could provide more binding sites due to the 3D surface (most assays are based on a 2D surface). Previous studies describe production of a zinc oxide (ZnO) nanowire (NW) on a glass substrate, which was labeled with fluorophores and a quantum dot (QD).^[^ ^16^, ^18^, ^19^ ^]^ The ZnO‐NW surface enhanced the fluorescence signal of the QD by about 8.5 times when compared with a bare glass substrate.^[^ ^19^ ^]^ Polymer nanofibers, silicon NW, nanostructured aluminum oxide, and ZnO‐NW have been utilized as substrates detection of biological molecules.^[^ ^15^, ^16^, ^17^, ^20^, ^21^, ^22^, ^24^, ^25^, ^26^ ^]^

In this study, we developed a novel microplate (ZnO‐NW MP) on which a ZnO NW was fabricated using a modified hydrothermal synthesis method; this was then used as the basis for an FIA to detect anti‐SARS‐CoV‐2 NP IgG antibodies. The ZnO‐NW MP bound high amounts of SARS‐CoV‐2 NP antigen tagged to histidine without any surface treatment. A high concentration (up to 5 µg mL^−1^) of SARS‐CoV‐2 NP antigen was attached to the ZnO‐NW MP when compared with the bare microplate. This serological immunoassay based on ZnO‐NW MP was more sensitive than a commercial assay, thereby facilitating early detection of anti‐SARS‐CoV‐2 NP antibodies in asymptomatic patients with COVID‐19.

## 2 Results and Discussion

### 2.1 Modified Hydrothermal Synthesis of ZnO‐NW on the MP

Nanowires can be fabricated using various methods; however, the most common methods are chemical vapor deposition (CVD) and the hydrothermal method. CVD requires a high temperature of around 1000 °C, which is much higher than the glass transition temperature (*T*_g_) and melting temperature of most polymers. ZnO‐NW can be fabricated simply under hydrothermal conditions that require a mild temperature (i.e., 90–95 °C). ZnO‐NW on substrates such as glass or silicon wafers has been synthesized using the hydrothermal synthesis method. However, it is hard to apply the NW to high throughput in vitro diagnostic (IVD) tests based on plasticware such as polystyrene, polypropylene, and polycarbonate, although ZnO‐NW has the potential for sensitive detection of biological targets, including antigens, antibodies, and genes.^[^ ^27^, ^28^, ^29^ ^]^

ZnO‐NW on a bare MP were synthesized using the convection heating method as reported previously (Figure S1a,b, Supporting Information^[^ ^28^ ^]^). This method resulted in nonuniformity of ZnO‐NW on the surface of each well, as well as high well‐to‐well variation (Figure S1c, Supporting Information). SEM analysis showed that the convection heating method also led to formation of undesirable micro‐scale structures on the ZnO‐NW (Figure S1c, Supporting Information). Detachment of ZnO‐NW was also observed during the immunoassay process such as (i.e., during incubation with solutions, and during the blocking and washing steps). The above method was not suitable for synthesizing ZnO‐NW on the MP to develop a sensitive immunoassay in a 96‐well MP. Therefore, a modified hydrothermal method to synthesis ZnO‐NW on a 96‐well microplate was designed and developed to improve both attachment and uniformity (Figure S1d, Supporting Information).

**Figure 1:**
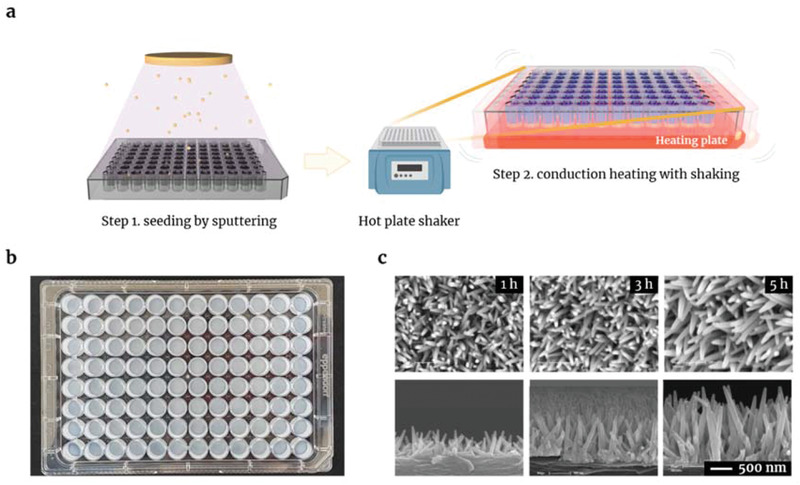
Modified hydrothermal synthesis of ZnO‐NW on a microplate. a) Schematic illustration of the modified ZnO‐NW synthesized using a hydrothermal method consisting of seeding by sputtering, followed by growing by conduction heating of ZnO‐NW precursor solution with shaking. b) Development of ZnO‐NW MP prepared by modified hydrothermal synthesis on a conventional polystyrene microplate (96‐well). c) SEM images of ZnO‐NW synthesized for 1, 3, and 5 h on the microplate.

This modified hydrothermal synthesis comprised two steps: seeding by sputtering (step 1) and growing by conduction heating and shaking (step 2). Step 1 effectively introduces nanoparticles onto the target surface (e.g., a polystyrene substrate); this step might also lead to uniform growth of ZnO‐NW precursors during the conduction heating step (**Figure** **1**). Replacement of fresh precursor solution during step 2 was required per every conduction heating step (over the 1‐h period) and was important for elimination of undesirable micro‐scale structures on the ZnO‐NW (Figure S1, Supporting Information). This modified hydrothermal synthesis generated the ZnO‐NW MP used in the study (Figure 1b). The morphological characteristics of the NW were examined by SEM (Figure 1c). The average diameter of NW grown for 1, 3, and 5 h was 63.5, 72.6, and 107.2 nm, respectively. The length was 550, 1011, and 1603 nm, respectively. Fluorescence signals were analyzed to investigate uniformity and well‐to‐well variation. The percent coefficient of variation (%CV) was 6.00, which was much less than that of NW grown by convection heating (%CV = 23.61; Figure S2, Supporting Information). Therefore, the ZnO‐NW MP prepared by our modified method should be suitable for immunoassays, including serological assay of COVID‐19.

### 2.2 Detection of SARS‐CoV‐2 NP Antibody on the ZnO‐NW Microplate

Previous research on ZnO‐NW‐based detection used surface treatment methods to immobilize biomolecules.^[^ ^19^ ^]^ Chemical reactions are commonly used to attach biomolecules to the surface of the ZnO‐NW. However, it takes hours to complete surface treatment, and the labor‐intensive process could increase the complexity of the assay. The properties of ZnO‐NW could be utilized to immobilize histidine‐tagged proteins; indeed, the affinity of histidine for Zn/ZnO has been investigated in previous studies.^[^ ^19^, ^30^, ^31^ ^]^ This property enables binding of antigens to the surface of the ZnO‐NW without pre‐preparation steps.

Binding tests were carried out using 6×His‐tag GFP and ZnO‐NW modified, or not, by (3‐aminopropyl) triethoxysilane (APTES) and glutaraldehyde (GA) treatment.^[^ ^19^ ^]^ There was no significant difference between no treatment and APTES modification of ZnO‐NW (data not shown). The binding of the 6×His‐tag GFP to the MP was also investigated in the presence/absence of ZnO‐NW. The GFP fluorescence signal in the ZnO‐NW MP was more than 10 times stronger than that in the bare MP. The GFP signals were enhanced differentially depending on the length of the conduction heating (1, 3, and 5 h) step used to grow ZnO‐NW precursors in each well of the 96‐well MP. Compared with the bare MP, the fluorescence signals were significantly enhanced by the conduction heating step for 1 and 3 h, respectively. However, there was no significant difference in signal enhancement between 3 and 5 h (Figure S3, Supporting Information). These results imply that 3 h of the conduction heating time may be appropriate for the enhancement of the GFP signals. According to all of above results, binding 6×His‐tag proteins to MP is increased by coating the wells with ZnO‐NW without requirement for chemical modification. This is likely due to the increase in surface area afforded by the ZnO‐NW. Therefore, we decided that the high binding ability of ZnO‐NW MP synthesized for 3 h is appropriate for development of a sensitive serological assay to detect SARS‐CoV‐2 antibodies.

The full XPS (X‐ray photoelectron spectroscopy) spectrum of ZnO‐NW was also analyzed to observe evidence in the presence of SARS‐CoV‐2 NP antigen on the ZnO‐NW MP. The characteristic peak of ZnO formation (Zn2P3/s) was observed at 1021 eV in both spectra (Figure S4, Supporting Information). Nitrogen (N) is one of the symbolic element of protein. The binding energy peak of nitrogen (N) 1s region was only observed in the SARS‐CoV‐2 NP‐coated condition of ZnO‐NW MP. Therefore, SARS‐CoV‐2 NP antigen was interfaced with the surface of the ZnO‐NW MP.

The large surface area of ZnO‐NW makes it useful for biosensing applications.^[^ ^19^ ^]^ The formation of a ZnO substrate for use in an immunoassay may provide more binding sites for biomolecules than conventional bare substrates such as MP. Here, we added NP 6×His‐tag antigen (1–10 µg mL^−1^) to the wells of the ZnO‐NW MP to determine the optimal concentration of NP for use in a serological assay for COVID‐19. Fluorescence intensity increased in a concentration‐dependent manner up to 5 µg mL^−1^ of NP antigen (Figure S5, Supporting Information). By contrast, there was no significant increase in the fluorescence signal when up to 1 µg mL^−1^ of NP antigen was incubated on the conventional bare MP (data not shown). Thus, ZnO‐NW MP has a higher binding capacity (at least 5 times) for his‐tagged proteins than the conventional bare MP. This property of ZnO‐NW MP should improve the sensitivity of a serological assay for COVID‐19.

**Figure 2:**
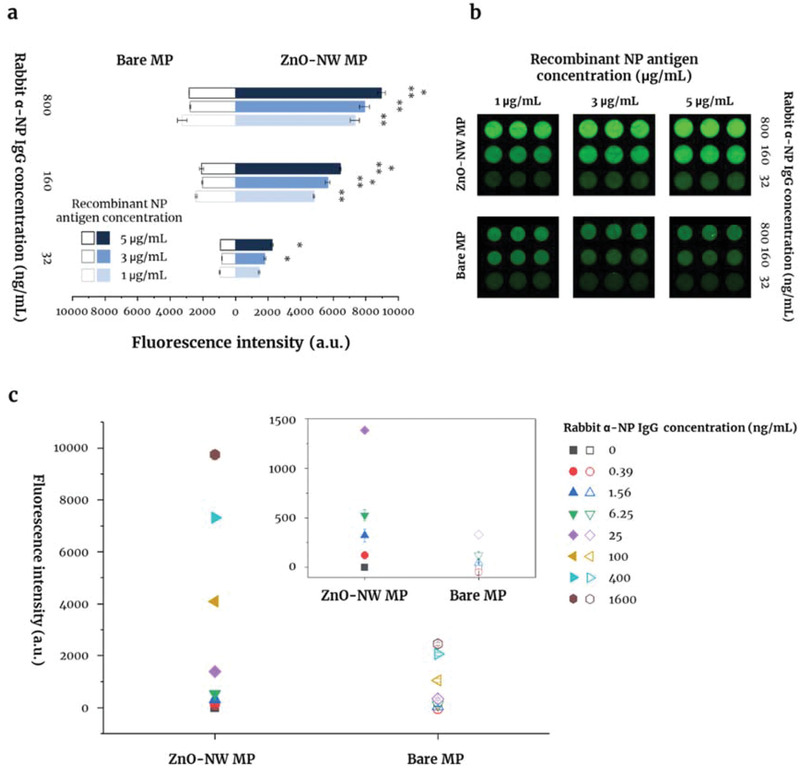
Introduction of SARS‐CoV‐2 NP antigen onto the surface of the ZnO‐NW MP, and detection of anti‐SARS‐CoV‐2 NP IgG polyclonal antibodies. a) SARS‐CoV‐2 NP antigen (1, 3, or 5 µg mL^−1^) was coated onto the ZnO‐NW MP and bare MP. Measurement of fluorescence intensity and imaging b) of the bound rabbit anti‐SARS‐CoV‐2 NP IgG polyclonal antibody (32, 160, or 800 ng mL^−1^) using anti‐human IgG conjugated to Alexa 488. c) Relative sensitivity of the ZnO‐NW MP coated with SARS‐Co‐2 NP (5 µg mL^−1^) for detection of SARS‐CoV‐2 NP IgG (inset plot: enlarged scale of fluorescence intensity from 0 to 1500 a.u.) . The data in the graphs are expressed as mean values ± SD. *p < 0.01 versus NP antigen at 1 µg/mL and the same antibody concentration, **p < 0.01 versus anti‐NP IgG at 32 ng mL^−1^ and the same antigen concentration.

Before using the ZnO‐NW MP for serological assay of COVID‐19, we conducted in vitro tests to compare binding of the SARS‐CoV‐2 NP antigen with that of a commercially available antibody. The fluorescence intensity of a rabbit anti‐SARS‐CoV‐2 NP IgG polyclonal antibody was tested by addition of anti‐human IgG conjugated to Alexa 488 to the ZnO‐NW MP after coating with NP (1, 3, or 5 µg mL^−1^) (**Figure** **2**). The difference in concentration (1, 3, or 5 µg mL^−1^) of NP antigen on the bare MP was not affected in the fluorescence intensity of anti‐SARS‐CoV‐2 NP IgG antibody (open bar in Figure 2a,b). The signals on the bare MP were not changed by the difference in concentration (32, 160, or 800 ng mL^−1^) of the antibody. On the contrary, the fluorescence intensity on the ZnO‐NW MP was significantly increased in a dose‐dependent manner of both NP antigen (1, 3, or 5 µg mL^−1^) and the antibody (32, 160, or 800 ng mL^−1^) concentration (closed bar in Figure 2a,b).

**Figure 3:**
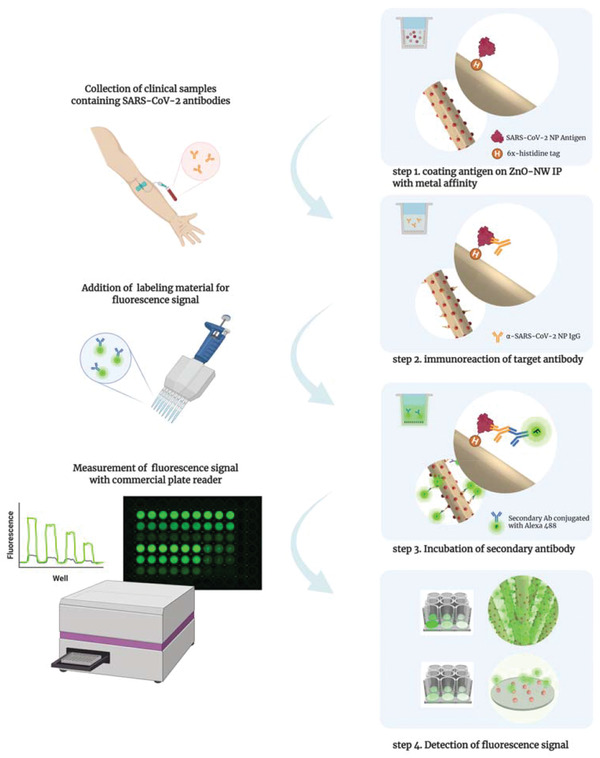
Schematic illustration of the method used to detect SARS‐CoV‐2 antibodies using the ZnO‐NW MP. ZnO‐NW MP‐based detection of SARS‐CoV‐2 antibody responses in COVID‐19 patients comprises four steps: coating of ZnO‐NW MP with SARS‐CoV‐2 NP antigen (step1); collection of patient blood sample and applying for immunoreaction with the target antibody (step 2); introduction of labeling materials and incubation with the secondary antibody conjugated to Alexa 488 (step 3); and detection of the fluorescence signal with a micro plate reader (step 4).

### 2.3 Study Population

The present study included 33 patients who tested positive for SARS‐CoV‐2 by RT‐qPCR. Some patients had clinical symptoms (*n* = 7) and some did not (*n* = 26). The median age of the patients in the clinical symptoms group was 61 years (interquartile range, 54.5–64.0) and that in the asymptomatic group was 66 years (interquartile range, 33.0–78.0). Six (85.7%) patients in the symptomatic group and 15 (57.7%) in the asymptomatic group were male. Among the clinical symptoms, fever was the most common (71.4%); other symptoms included cough (42.9%), myalgia (57.1%), anosmia and/or dysgeusia (28.6%), and dyspnea (14.3%). Underlying diseases included hypertension, diabetes mellitus, heart disease, cerebrovascular disease, chronic renal failure, and solid tumors. The incidence of underlying disease was not significantly different between the two groups. The demographic and laboratory findings of the enrolled patients are summarized in **Table** **1**.

**Table 1:** Demographics and clinical characteristics of symptomatic and asymptomatic patients who tested positive for SARS‐CoV‐2 by RT‐PCR

Variable	Symptomatic patients		Asymptomatic patients	
	(*n* = 7)	0–5 d (*n* = 8)	(*n* = 26)	0–5 d (*n* = 34)
Age, median (IQR^a)^)	61 (54.5–64)		66 (33–78)	
Male sex, *n* [%]	6 (85.7)	7 (87.5)	15 (57.7)	21 (61.8)
RT‐PCR analysis				
*C*_t_ ^b)^ values, *n*, mean ± SD	7, 24.92 ± 5.1		19, 25.07 ± 6.53	
Interval from initial confirmation to follow‐up RT‐PCR test; *n*, median (IQR)	7, 1 (0–4.5)		19, 1 (0–1.25)	
Symptoms, *n* [%]				
Fever	5 (71.4)	6 (75)	0	0
Cough	3 (42.9)	3 (37.5)	0	0
Dyspnea	1 (14.3)	1 (12.5)	0	0
Anosmia and/or dysgeusia	2 (28.6)	2 (25)	0	0
Myalgia	4 (57.1)	4 (50)	0	0
Underlying diseases				
Diabetes mellitus	2 (28.6)	2 (25)	2 (7.7)	3 (8.8)
Hypertension	1 (14.3)	1 (12.5)	7 (26.9)	10 (29.4)
Cerebrovascular disease	0 (0)	0 (0)	1 (3.8)	2 (5.9)
Heart diseases	0 (0)	0 (0)	2 (7.7)	2 (5.9)
Chronic renal failure	1 (14.3)	1 (12.5)	1 (3.8)	1 (2.9)
Solid tumors	0 (0)	0 (0)	4 (15.4)	5 (14.7)
Laboratory tests, *n*, mean ± SD				
White blood cell [×10^3^, µL]	7, 5.02 ± 1.16	8, 5.90 ± 2.59	26, 5.36 ± 1.82	34, 5.28 ± 1.76
Erythrocyte sedimentation rate [mm h^−1^]	7, 31.86 ± 20.91	7, 31.86 ± 20.91	19, 42.05 ± 50.14	21,39.14 ± 48.55
C‐reactive protein [mg L^−1^]	7, 36.18 ± 34.38	8, 33.33 ± 33.03	26, 15.92 ± 37.5	34, 15.26 ± 34.32
Platelets [×10^3^, µL]	7, 169.14 ± 40.16	8, 165.25 ± 38.95	26, 206.81 ± 71.4	34, 207.53 ± 70.58
Aspartate aminotransferase [IU L^−1^]	7, 41.71 ± 18.48	8, 40.25 ± 17.71	26, 26.54 ± 9.53	34, 27.53 ± 13.06
Alanine aminotransferase [IU L^−1^]	7, 35.71 ± 16.26	8, 33.63 ± 16.26	26, 21.62 ± 16.1	34, 23.50 ± 19.13
Total bilirubin [mg mdL^−1^]	7, 0.70 ± 0.26	8, 0.74 ± 0.27	26, 0.56 ± 0.26	34, 0.60 ± 0.30
Creatinine [mg dL^−1^]	7, 0.91 ± 0.26	8, 0.86 ± 0.28	26, 1.33 ± 2.38	34, 1.46 ± 2.50

^a)^ Interquartile range;

^b)^ cycle threshold.

### 2.4 Early Detection of SARS‐CoV‐2 Antibody Responses in Asymptomatic Patients with COVID‐19

**Figure 4:**
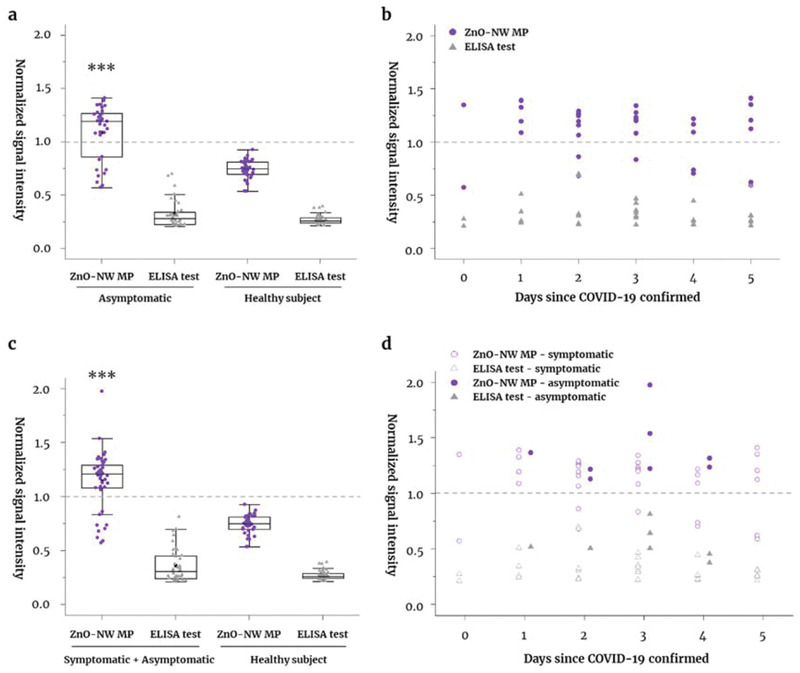
Detection of SARS‐CoV‐2 antibody responses in symptomatic and asymptomatic individuals with COVID‐19. SARS‐CoV‐2 antibody responses were analyzed using the ZnO‐NW MP‐based serological assay and with a conventional ELISA test for a) symptomatic and b) asymptomatic patients (within < 5 days of a PCR‐confirmed infection) with COVID‐19. Data were compared with those for healthy subjects. Comparison of results from daily (0−5 days) tests of samples from c) symptomatic and d) asymptomatic patients; tests were carried out using the ZnO‐NW MP‐based serological assay and the conventional ELISA test. Median values and cut‐offs are denoted by solid line in the box and dotted lines in the box plot, respectively. Data on the graphs are expressed as mean values ± SD. ***p < 0.0001 versus healthy subjects.

Next, we used the ZnO‐NW MP to detect antibodies in clinical samples from symptomatic and asymptomatic patients with COVID‐19 (**Figure** **3**). All of these samples in the present study were within 5 days after COVID‐19 confirmed by RT‐qPCR. The ZnO‐NW MP‐based serological assay returned positive results for 100% (*p* < 0.0001) of symptomatic patients (*n* = 8). There were no positive results for specimens (*n* = 40) from healthy subjects (all of whom were RT‐qPCR‐negative for COVID‐19). By contrast, the conventional ELISA test for anti‐SARS‐CoV‐2 NP IgG returned no positive results (**Figure** **4**a). The ZnO‐NW MP‐based serological assay detected antibodies within 1–4 days of since confirmation of COVID‐19. However, the conventional ELISA test did not return a positive result within the same period (Figure 4b). This implies that ZnO‐NW MP is a more sensitive serological assay for detection of SARS‐CoV‐2 NP IgG antibodies than the conventional ELISA test, particularly during the early phase (<5 days) after infection.

A wide range of serological assays (e.g., chemiluminescent immunoassay (CLIA), enzyme immunoassay (EIA), lateral flow immunoassay (LFIA), and microsphere‐based antibody assays) have been developed to detect SARS‐CoV‐2 antibodies.^[^ ^32^, ^33^ ^]^ These assays are based on a bilateral interaction between SARS‐CoV‐2 antigens (S protein, receptor‐binding domain, and NP) and isotype‐specific antibodies (IgG, IgA, or IgM). The performance of various serological assays has been compared using clinical samples from symptomatic patients with COVID‐19. These serological assays have been poor with respect to detecting an early immune response and seroconversion in symptomatic patients.^[^ ^4^, ^9^, ^13^ ^]^ Early detection of SARS‐CoV‐2 antibody responses in asymptomatic patients has not yet been reported.

When we used the ZnO‐NW MP assay to investigate clinical samples from asymptomatic patients with COVID‐19, we found that it returned positive results in 73.5% of cases (*n* = 25) with a RT‐qPCR‐confirmed infection. By contrast, the conventional ELISA test returned no positive results (Figure 4c). In addition, the ZnO‐NW MP‐based serological assay was positive for antibodies within 0–2 days and 3–5 days of a confirmed COVID‐19 infection (80% and 68.4%, respectively (Figure 4d). Total serology results from symptomatic (*n* = 8) and asymptomatic (*n* = 34) COVID‐19 patients (total = 42), and those from healthy subjects, are summarized in **Table** **2**. The ZnO‐NW MP‐based assay returned positive results in 78.6% (*p* < 0.0001) of all cases (95% confidence interval, 63.2–89.7), whereas the conventional ELISA test returned none. Therefore, ZnO‐NW MP is a sensitive method for early detection of SARS‐CoV‐2 antibody responses in asymptomatic patients with COVID‐19.

**Table 2:** Summary of serological tests from symptomatic and asymptomatic patients

	Symptomatic patient				Asymptomatic patient				Total patient			
	Total *n*	Positive *n*	Positive [%]	95% CI^a)^	Total *n*	Positive *n*	Positive [%]	95% CI	Total *n*	Positive *n*	Positive %	95% CI
ZnO‐NW MP	8	8	100	63−100	34	25	73.5	55.6−87.1	42	33	78.6	63.2−89.7
ELISA test	8	0	0	0−36.9	34	0	0	0−10.3	42	0	0	0−0. 08

^a)^ Confidence interval.

## 3 Conclusion

During the COVID‐19 pandemic, the detection of antibodies specific for viral antigen has become important to get a more accurate estimate of the prevalence and incidence of SARS‐CoV‐2. We developed a novel ZnO NW‐based serological assay for sensitive and early detection of SARS‐CoV‐2 antibody responses. This serological assay was able to detect early antibody responses in asymptomatic patients with COVID‐19 as well as symptomatic patients. To the best of our knowledge, this is the first report of a sensitive serological assay capable of detecting early antibody responses to SARS‐CoV‐2 in asymptomatic patients with a confirmed diagnosis of COVID‐19. The ZnO‐NW MP assay has the potential for high throughput, making it useful for rapid and accurate diagnosis of COVID‐19, as well as for estimation of its prevalence and incidence. Moreover, ZnO NW‐based immunoassay could be adapted for serological test of other emerging infectious diseases.

## 4 Experimental Section

### Chemicals and Reagents

The ZnO nanowire hydrothermal synthesis method was modified and applied to conventional plastic‐based well plates.^[^ ^28^ ^]^ Briefly, a ZnO‐NW precursor was prepared from zinc nitrate hexahydrate (Zn(NO_3_)_2_·6H_2_O, 228737, Sigma‐Aldrich, USA) and hexamethylenetetramine (C_6_H_12_N_4_, 398160, Sigma‐Aldrich, USA). A fluorescence signal was generated by conjugation of histidine‐tagged GFP (Aequorea Victoria GFP His‐tag recombinant protein; A42613, Invitrogen, USA) to the surface of ZnO‐NW. Cloning, expression, and purification of SARS‐CoV‐2 NP with an N‐terminal 6 × His‐tag were performed as previously described.^[^ ^3^ ^]^ Briefly, the full‐length SARS‐CoV‐2 NP gene in a pET28a vector was transformed into *E. coli* BL21(DE3). Soluble NP was purified using Ni^2+^ affinity and size‐exclusion chromatography after induction of bacterial cells by treatment with 0.5 × 10^−3^ m isopropyl β‐d‐1‐thiogalactopyranoside. The purity of the recombinant NP was examined by sodium dodecyl sulfate‐polyacrylamide gel electrophoresis (SDS‐PAGE). A rabbit polyclonal antibody specific for SARS‐CoV‐2 NP was obtained for use as a target molecule (40588‐T62; SinoBiological, China). Alexa fluor 488 conjugated anti‐rabbit IgG (A‐11034, Invitrogen, USA) and anti‐human IgG (A‐11013, Invitrogen, USA) were used to generate a fluorescence signal. Human serum (H4522, Sigma‐Aldrich, USA,) was purchased from Sigma‐Aldrich and used as a negative (healthy) control for sensitivity test.

### Hydrothermal Synthesis of Modified ZnO‐NW on a 96‐well Plate

The wells of a polystyrene 96 microplate (0030730011, Eppendorf, Germany) were coated with a thin film of ZnO using a sputtering process. The sputtering was performed at 300 W for 10 min. The ZnO‐NW was synthesized hydrothermally by heating (90 °C) and shaking (500 rpm) the plate with the ZnO‐NW precursor in a hot plate shaker (ThermoMixer C, Eppendorf, Germany) for 1–5 h (Figure 1a). The NW‐grown 96‐well microplate was then washed with deionized water using an automatic washer dispenser (EL406, BioTek, USA), followed by air‐blow drying and oven‐drying at 60 °C for 1 h (Figure 1a,b).

### Characterization of the ZnO‐NW Microplate

The grown NW structure was observed under a scanning electron microscope (SEM) (Figure 1c). The diameter and length of the nanowires were measured on the SEM images. The signal enhancement effect was confirmed by conjugation of histidine‐tagged GFP. Briefly, His‐tag GFP was prepared in coating buffer A (CB07100, Invitrogen, USA) at a final concentration of 10 µg mL^−1^. Next, 100 µL of the solution was introduced into each well coated with nanowires grown for 1, 3, or 5 h. A bare MP, a conventional immunoassay plate (Nunc Maxisorp, 44‐2404‐21, Invitrogen, USA), was used as a control to compare the signal enhancement efficiency after morphological changes in the nanowire. After incubation for 1 h, the plate was washed with ELISA wash buffer (WB01, Invitrogen, USA) in an automatic washer dispenser. The fluorescence signal was measured in a conventional microplate reader (Synergy H1, BioTek, USA) at excitation and emission wavelengths of 485 and 528 nm. XPS data were analyzed Thermo Fisher Scientific K‐Alpha+ XPS using a monochromatic Al Kα radiation (15 kV, 15 mA). Binding energies were corrected by referencing the C (1s) binding energy to 285 eV.

### Determination of the Antigen Concentration in the ZnO‐NW Microplate

A polyclonal anti‐SARS‐CoV‐2 NP was detected after direct interaction with the NP antigen coated onto the surface of the ZnO‐NW MP. A bare MP was used as a control. Quantification was performed by measuring the fluorescence signal generated by a fluorescence‐conjugated secondary antibody, which reacted with the target antibody. First, the optimum amount of NP antigen was determined. To do this, NP antigen in coating buffer A (CB07100, Invitrogen, USA) was prepared at 1, 3, or 5 µg mL^−1^ and added to the ZnO‐NW MP. Next, the anti‐NP polyclonal antibody (32, 160, or 800 ng mL^−1^) in assay buffer (DS98200, Invitrogen, USA) was applied to the plate for 1 h, followed by the Alexa Fluor 488‐conjugated secondary antibody (A‐11034; 1 mg mL^−1^) for a further hour. The plate was washed between these steps. The fluorescence signal was measured in a microplate reader and an image taken under a laser scanning imager (Sapphire Biomolecular Imager, Azure biosystems, USA).

### Sensitivity of the ZnO‐NW MP for Anti‐SARS‐CoV‐2 NP Antibodies

The ZnO‐NW MP was prepared with NP antigen coating method described above. The bare MP was prepared using the same protocol. After antigen coating, each plate was washed in a washer dispenser and then blocked with 300 µL of blocking buffer for 1 h. The anti‐SARS‐CoV‐2 NP polyclonal antibody was serially diluted 2‐fold from 3200 to 0.39063 ng mL^−1^. Next, 100 µL of antibody solution was introduced into the wells of the plates and incubated for 1 h. This was followed by washing and addition of an Alexa 488‐labeled secondary antibody (1 µg mL^−1^) for a further 1 hour. The fluorescence signal was measured as described above.

### Preparation of Clinical Samples

Serum (*n* = 42) and upper respiratory tract specimens (naso‐ and oropharyngeal swabs, *n* = 26) from COVID‐19 patients (*n* = 33) and healthy subjects (*n* = 40) were collected in accordance with the registered protocols approved by the Institutional Review Board (IRB) of Jeonbuk National University Hospital. All patients provided written informed consent (IRB registration number: CUH2021‐06‐036‐002). The upper respiratory tract samples were collected in a universal transport medium (UTM; Noble Bio, Hwaseong, Republic of Korea). Serum samples were prepared from whole blood samples from COVID‐19 patients and healthy subjects.^[^ ^4^ ^]^ All clinical samples were sub‐aliquoted and stored at −80 °C until further analysis. Each aliquot of serum was inactivated by heating at 56 °C for 1 h.^[^ ^4^ ^]^

### RT‐qPCR Analysis

Nasopharyngeal swabs from COVID‐19 patients (symptomatic, *n* = 7; asymptomatic, *n* = 19) were collected and stored in the transport medium (eNAT; COPAN, USA) for viral RNAs. All clinical samples were subsequently inactivated by heating at 100 °C for 10 min and then stored at −80 °C until use. Viral RNA was purified from 70 µL of clinical sample using a QIAamp viral RNA extraction kit (52906, Qiagen, Germany). The extracted RNA was amplified using the Luna universal probe one‐step RT‐qPCR kit (E3006L, New England Biolabs Inc., USA). Briefly, 20 µL of reaction mixture contained 1× Luna universal probe one‐step reaction mix, 1 µL of Luna WarmStart RT enzyme mix, 400 × 10^−9^ m of primers, 200 × 10^−9^ m of probes, and 1 µL of viral RNA. The primer‐probe set used in this study was “NIID_2019‐nCOV_N” from the Japan NIID, which is recommended for N gene amplification.^[^ ^1^ ^]^ RT‐qPCR was performed in a CFX 96 touch real‐time PCR detection system (Bio‐Rad, USA) under the following conditions: 10 min at 55 °C for reverse transcription, 1 min at 95 °C for initial denaturation, followed by thermal cycling at 95 °C for 10 s (denaturation) and 60 °C for 30 s (extension) (total, 45 cycles). *C*_t_ values for triplicate samples from each patient are expressed as the mean value calculated from three separate reactions.

### Serological Assay with COVID‐19 Specimens

For serological assay of anti‐SARS‐CoV‐2 NP IgG from COVID‐19 patient serum, the ZnO‐NW MP was prepared and blocked as described above. Next, 100 µL of diluted sample (1:1000 in assay buffer) from COVID‐19‐positive patients with/without symptoms were applied to each well and incubated at room temperature for 1 h. After washing, the Alexa 488‐labeled secondary antibody (1 µg mL^−1^) was added to each well for 1 hour. The fluorescence signal was detected as described above. To compare the efficiency of the ZnO‐NW MP, the same diluted samples were tested using a commercially available ELISA kit (KT‐1032, Epitope Diagnostics, Inc., USA).

### Statistical Analysis

OriginPro v. 2021b (OriginLab, Northampton, Massachusetts, USA) was used for statistical analysis. All data are expressed as the mean ± SD. A *p*‐value < 0.05 was regarded as significant. One‐way ANOVA followed by Tukey's test was used to evaluate differences between more than two groups.

## Conflict of Interest

The authors declare no conflict of interest.

## Supporting information

Supporting Information

## Data Availability

The data that support the findings of this study are available on request from the corresponding author upon reasonable request. The data are not publicly available due to privacy or ethical restrictions.
