# Supplementary material for: ZnO Nanowire‐Based Early Detection of SARS‐CoV‐2 Antibody Responses in Asymptomatic Patients with COVID‐19
Source: Adv Mater Interfaces. 2022 Feb 5;9(14):2102046. doi: 10.1002/admi.202102046 (PMC9073923; doi:10.1002/admi.202102046)
Supplement: Supplementary file 1 — Supporting Information [file ADMI-9-0-s001.pdf]

ADVANCED MATERIALS INTERFACES

Supporting Information

for *Adv. Mater. Interfaces,* DOI: 10.1002/admi.202102046

ZnO Nanowire-Based Early Detection of SARS-CoV-2 Antibody Responses in Asymptomatic Patients with COVID-19

Jung Kim, Sung Kyun Lee, Jong-Hwan Lee, Hye-Yeon Kim, Nam Hoon Kim, Chang Hoon Lee, Chang-Seop Lee, and Hong Gi Kim**

Supplementary information

ZnO nanowire-based early detection of SARS-CoV-2 antibody responses in asymptomatic patients with COVID-19

Jung Kim[1], Sung Kyun Lee[1], Jong-Hwan Lee[1], Hye-Yeon Kim[1, 2], Nam Hoon Kim[1], Chang Hoon Lee[3], Chang-Seop Lee[4,], and Hong Gi Kim[1,*]*

J. Kim, S. K. Lee, J. –H. Lee, H. –Y. Kim, N. H. Kim, H. G. Kim

[1]Center for Convergent Research of Emerging Virus Infection, Korea Research Institute of Chemical Technology, Daejeon 34114, Republic of Korea

H. –Y. Kim

[2]Research Center for Bioconvergence Analysis, Korea Basic Science Institute, Cheonju, 28119, Republic of Korea

C. H. Lee

[3]Drug Discovery Platform Research Center, Therapeutic & Biotechnology Division, Korea Research Institute of Chemical Technology, Daejeon 34114, Republic of Korea

C. –S. Lee

[4]Department of Internal Medicine, Jeonbuk National University Medical School, Jeonju, Jeollabuk-do 54986, Republic of Korea; Biomedical Research Institute of Jeonbuk National University Hospital, Jeonju, Jeollabuk-do 54907, Republic of Korea

Keywords: COVID-19, Asymptomatic, Antibody response, early detection, ZnO nanowire

* Corresponding authors: Chang-Seop Lee (lcsmd@jbnu.ac.kr) and Hong Gi Kim (tenork@krict.re.kr)

Supplementary materials and methods

Comparison of Convection and Conduction Heating for Hydrothermal Synthesis of ZnO-NW

ZnO-NW was synthesized on the bare microplate using the convection heating and conduction heating methods. A thin layer of ZnO and ZnO-NW precursor was prepared as described in 2.2. For convection hydrothermal synthesis, the prepared microplate was immersed upside down in the precursor and heated in the convection oven for 3 hours at 95°C. The ZnO-NW MP was prepared as described in 2.2. To evaluate surface uniformity precisely, an auto-fluorescence signal was observed at 485 and 528 nm (excitation and emission, respectively).

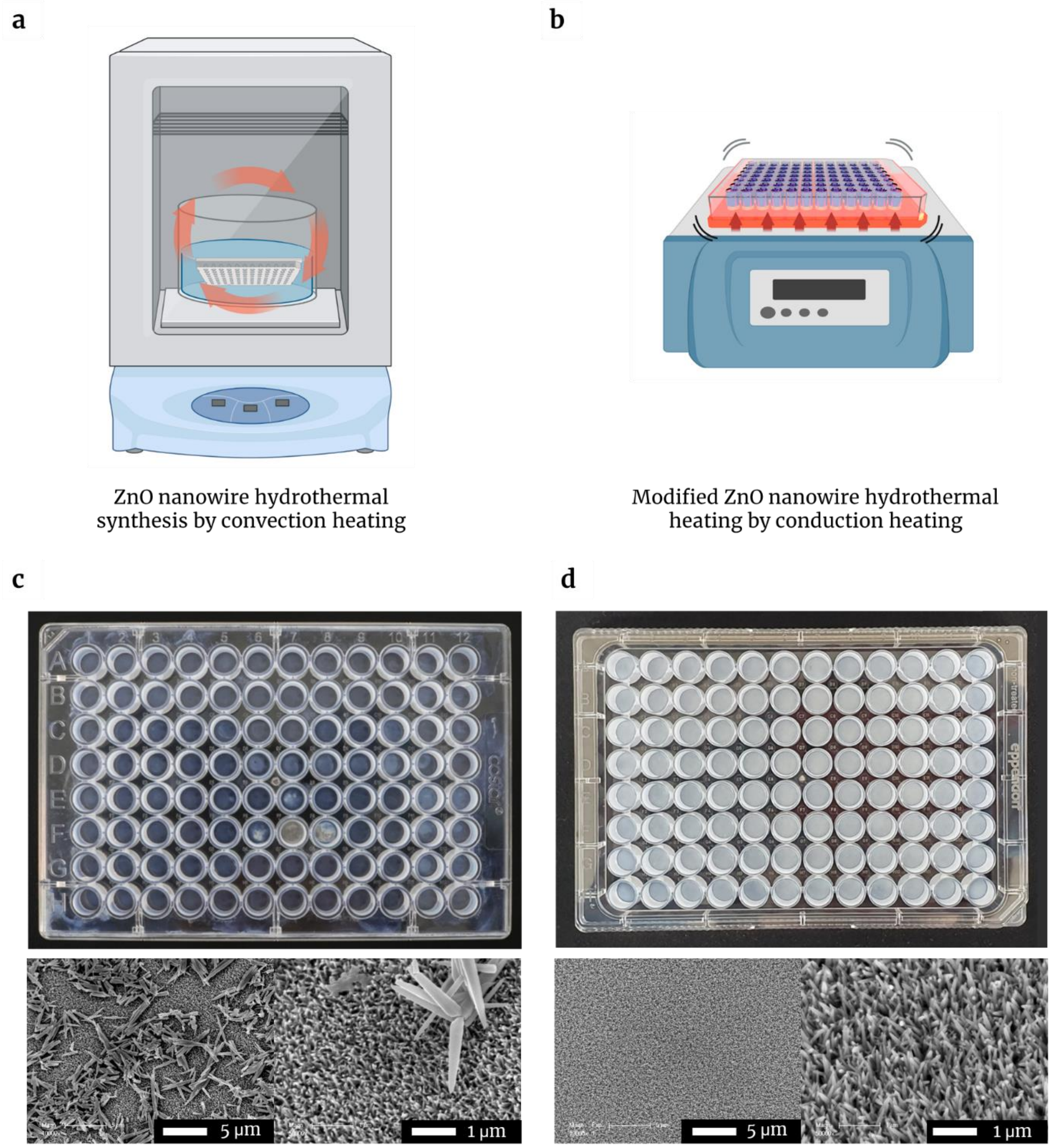

Figure S1. Comparison of ZnO-NW synthesized by the convection and conduction heating methods. (a) The convection heating ZnO-NW hydrothermal synthesis method and (b) the conduction heating method. (c) Photos of ZnO-NW MP and SEM images of ZnO-NW synthesized by the convection heating method and (d) the conduction heating method.

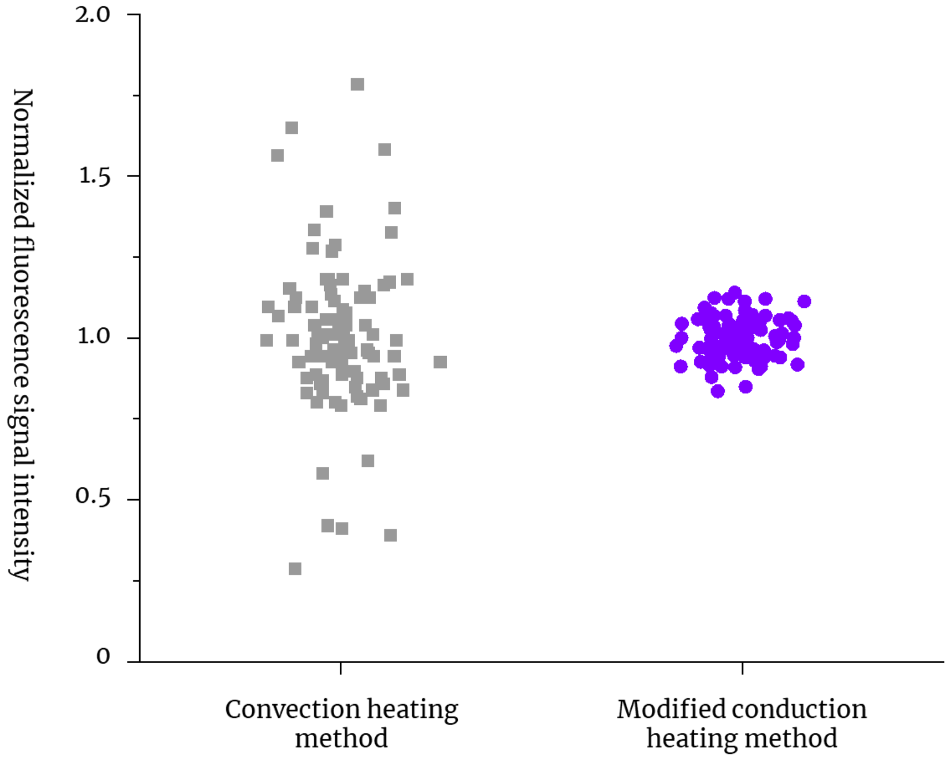

Figure S2. Comparison of fluorescence signals generated after the convection heating and conduction heating methods. The fluorescence signal in each well was measured at 485 and 528 nm (excitation and emission, respectively). The percent coefficient of variation (%CV) for well-to-well variation was calculated using fluorescence the signals from each well of ZnO-NW MP. The %CV of the fluorescence signals from all wells of the ZnO-NW MP constructed by the convection heating method was 23.61, compared with 6% for the conduction heating method.

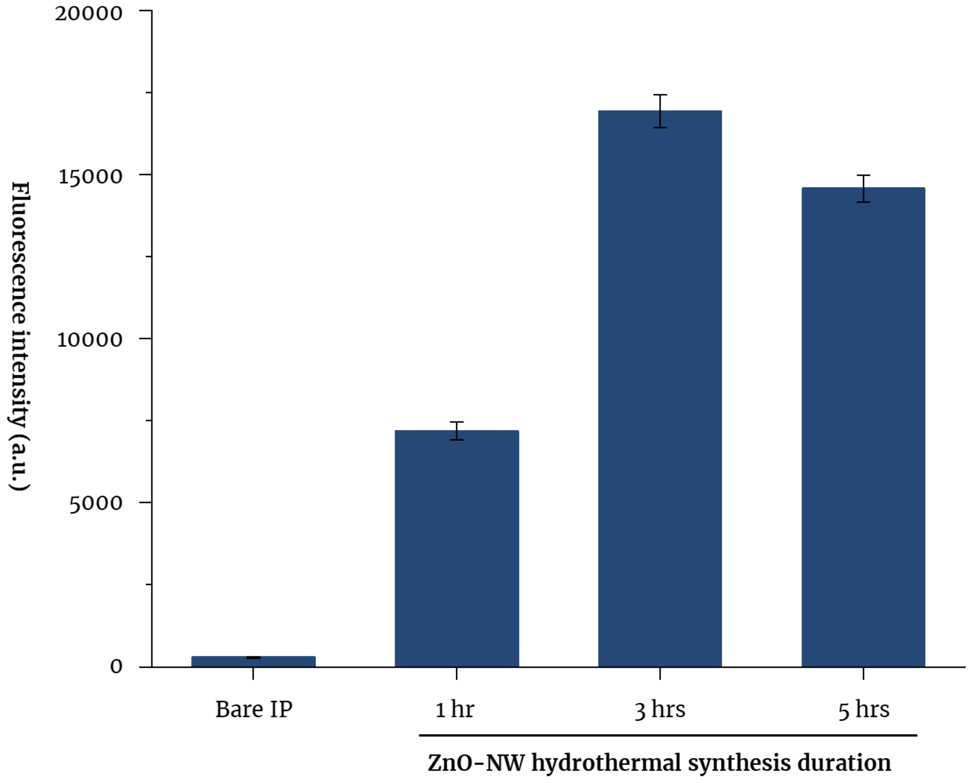

Figure S3. The binding of 6xHis-tag GFP to grown ZnO-NW at different times. The average GFP signal intensity in wells containing ZnO-NW grown for 1, 3, and 5 hours was 7179, 16926, and 14571, respectively. Data in the graph are expressed as mean values ± SD. *$p < 0.01$ *vs*. bare MP.

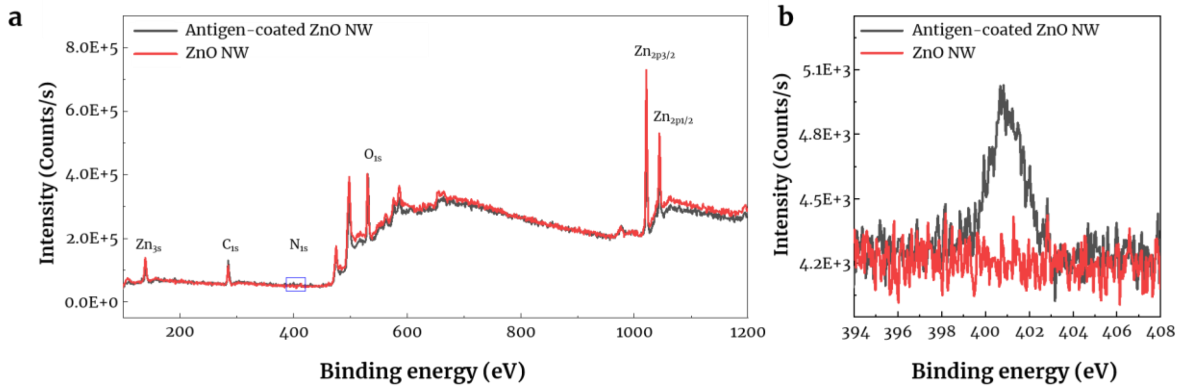

Figure S4. The full range XPS spectrum and high resolution XPS spectra N1s of ZnO NW in the presence of SARS-CoV-2 NP antigen. (a) Major peaks including Zn, C, N, and O formation were observed in the full range XPS spectrum. (b) The binding energy peak of nitrogen (N) 1s region was only observed in the SARS-CoV-2 NP-coated condition of ZnO-NW MP.

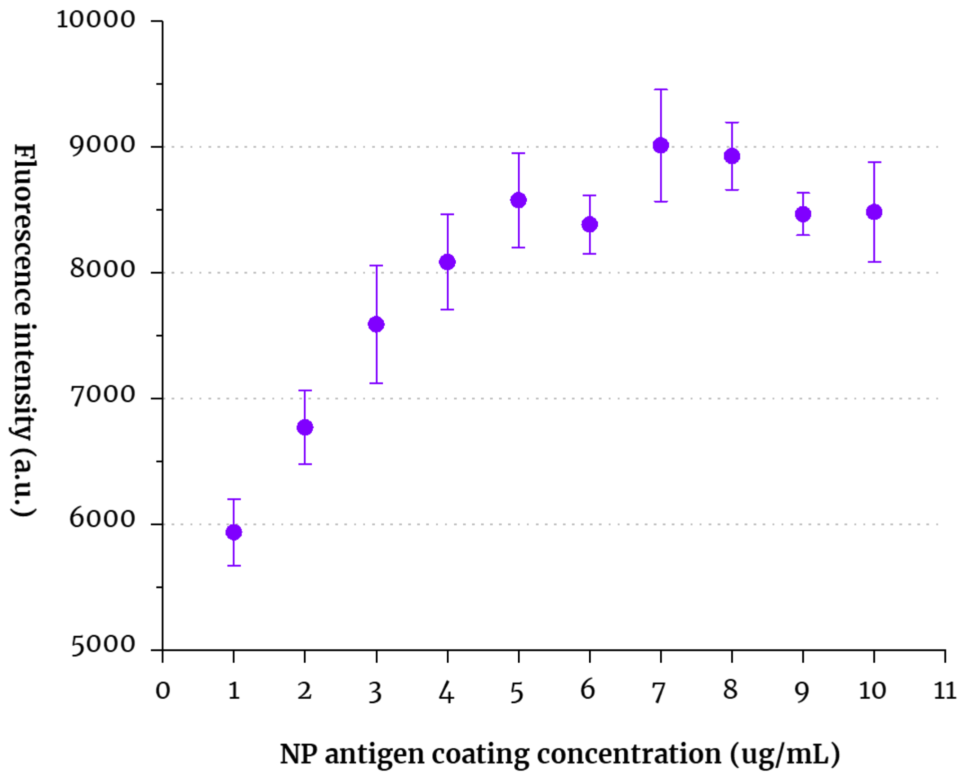

Figure S5. Determination of the optimal antigen concentration for the SARS-CoV-2 NP serological assay using ZnO-NW MP. SARS-CoV-2 antigen (1–10 μg/mL) was coated onto the wells of the ZnO-NW MP. Fluorescence intensity was measured after binding of an anti-rabbit antibody conjugated to Alexa 488 to a rabbit anti-SARS-CoV-2 NP IgG antibody. Data in the graph are expressed as mean values ± SD.
